# Hearing Loss: Genetic Testing, Current Advances and the Situation in Latin America

**DOI:** 10.3390/genes15020178

**Published:** 2024-01-29

**Authors:** Maria Agustina De Rosa, Maria T. Bernardi, Soledad Kleppe, Katherina Walz

**Affiliations:** 1 Instituto de Química Biológica de la Facultad de Ciencias Exactas y Naturales (IQUIBICEN) CONICET, Facultad de Ciencias Exactas y Naturales, Universidad de Buenos Aires, Buenos Aires C1428EHA, Argentina; magustinaderosa@gmail.com (M.A.D.R.); mariat.bernardi@gmail.com (M.T.B.); 2 Department of Clinical Pediatrics, Hospital Italiano de Buenos Aires, Instituto Universitario Hospital Italiano de Buenos Aires, Buenos Aires C1199ABB, Argentina; soledad.kleppe@hospitalitaliano.org.ar; 3 John P. Hussman Institute for Human Genomics, Miller School of Medicine, University of Miami, Miami, FL 33136, USA; 4 John T. Macdonald Foundation Department of Human Genetics, Miller School of Medicine, University of Miami, 1501 NW 10th Avenue, BRB-418 (M-860), Miami, FL 33136, USA

**Keywords:** sensorineural hearing loss, genetics, diagnosis, Latin America

## Abstract

Congenital hearing loss is the most common birth defect, estimated to affect 2–3 in every 1000 births, with ~50–60% of those related to genetic causes. Technological advances enabled the identification of hundreds of genes related to hearing loss (HL), with important implications for patients, their families, and the community. Despite these advances, in Latin America, the population with hearing loss remains underdiagnosed, with most studies focusing on a single locus encompassing the *GJB2*/*GJB6* genes. Here we discuss how current and emerging genetic knowledge has the potential to alter the approach to diagnosis and management of hearing loss, which is the current situation in Latin America, and the barriers that still need to be overcome.

## 1. Introduction

Hearing loss (HL) affects ~30% of the world population at some time in their lives. Clinically significant HL is present in ~2 per 1000 newborns in the U.S. [1] rising to at least 2.7 per 1000 by four years of age. According to the World Health Organization (WHO), more than 1.5 billion people (~20% of the global population) currently live with HL, and more than 5% of the world’s population—~430 million people—need rehabilitation. Estimations indicate that by 2050, those numbers will rise to ~2.5 billion people having some degree of hearing loss and ~700 million (or 1 in 10 individuals) requiring rehabilitation [2]). HL is a major contributor to years lived with disability (YLDs) globally, for both sex and at all ages, negatively affecting well-being [3]. 

HL is a condition of diverse presentation and etiology. It can be classified by severity, by the level of hearing below the normal threshold measured in decibels (dB). A decrease of up to 20 dB is still considered to be normal, a decrease of 26–40 dB is classified as mild, 41–55 dB is moderate, 56–70 dB is moderately severe, 71–90 dB is severe and >90 dB is profound [4]. The classification of hearing impairment can be performed with the topographic and functional abnormality. Conductive HL (CHL) is characterized by external ear anomalies or abnormalities of the ossicles in the middle ear. Sensory HL (SHL) is related to dysfunction of the organ of Corti. Neural HL (NHL) is due to diseases affecting the cochlear nerve (including its synapses), and Sensorineural HL (SNHL) is related to the combination of both. In addition, central hearing loss is caused by defects of the VIIIth nerve, the brain stem, or the cerebral cortex. Of course, HL can be associated with a mix of the mentioned causes [5]. HL is also classified as progressive or non-progressive if the onset is before (prelingual) or after (post-lingual) verbal communication; syndromic or non-syndromic if it appears alone or with other symptoms (30% and 70% of cases, respectively); and bilateral or unilateral, affecting both or only one ear [6]. 

There are many known environmental and genetic causes of deafness. Environmental factors include prematurity, hyperbilirubinemia, very low weight at birth, congenital infections with agents such as rubella or cytomegalovirus, meningitis, severe chronic otitis media, head trauma, pharmacologic ototoxicity, and exposure to loud or constant noises (e.g., the use of headphones at high volume that is increasing the incidence of hearing loss) [7], with many of these cases being preventable. Genetic factors account for ~50–60% of congenital HL cases [1,8,9], and inheritance can be autosomal recessive (77%), dominant (22%), and X-linked (1%) [10]. Genetic cases of deafness are mostly SNHL [11,12]. 

## 2. Genetic Discoveries Related to SNHL

**Figure 1:**
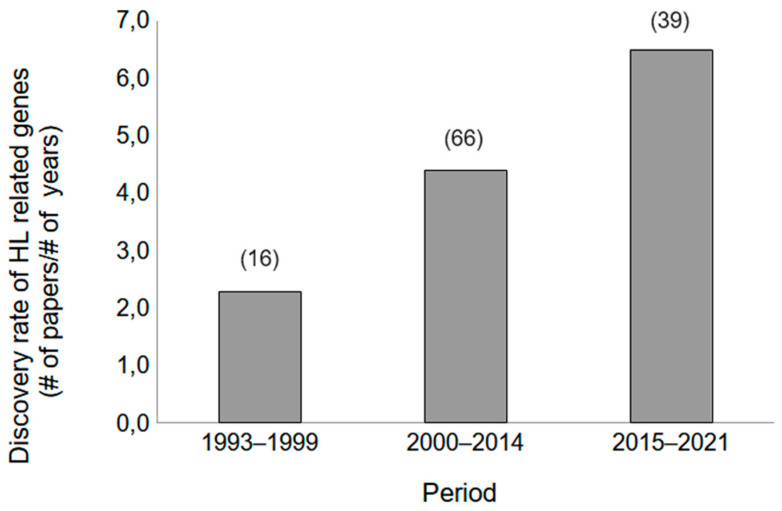
The gene discovery rate for SNHL. The curated list of genes related to hearing loss https://hereditaryhearingloss.org (accessed on 26 January 2024), was used to analyze the rate of HL-related genes discovered/year (# means number). We defined three different periods, with the beginning of each period coinciding with a specific landmark. Period 1 (6 years) began with the discovery of the first SNHL gene in 1993, Period 2 (15 years) began with the publication of the human reference genome in 2000, and Period 3 (7 years) began with the cost per human genome reaching USD~1000 in 2015 and ending in 2021. The total number of genes discovered in each period is shown in brackets.

Most congenital deafness is monogenic, but the high degree of genetic heterogeneity made gene discovery a huge challenge. The rate of gene discovery can be divided into three periods, coinciding with important landmarks as cutoffs (1) the discovery of the first gene-related SNHL (2) the publication of the human genome, and (3) the cost per human genome reaching USD~1000 (Figure 1). In 1993 Prezant et al. [13] described a nucleotide 1555 A to G substitution in the 12S rRNA gene, related to non-syndromic sensorineural deafness and susceptibility to aminoglycoside ototoxicity. Interestingly this site is implicated in aminoglycoside activity [14]. In 1995, cases of progressive mixed HL, (conductive and sensorineural) and characteristic bone anomalies were associated with sequence variations in the POU domain of the BRAIN-4 (*POU3F4*, OMIM# 300039) gene [15]. The *POU3F4* gene located in the Xq21 band, encodes for a transcription factor [16,17,18] and is the most common X-linked form of HL and despite low overall incidence, the discovery was facilitated by clear mapping and linkage analysis. In 1997, mutations at a single locus, DFNB1 (OMIM # 220290), accounting for 30–40% of non-syndromic sensorineural hearing loss in many populations was discovered [19,20]. DFNB1 comprises the *GJB2* and *GJB6* genes, encoding for connexins 26 and 30, both subunits of gap junction proteins expressed in the inner ear forming channels between adjacent cells for small molecule exchange. In addition, large deletions spanning the *GJB6* gene can cause deafness when present in trans with a single pathologic *GJB2* mutation [21,22,23,24]. Currently, the deletions at the *GJB6* gene are associated with the regulatory region of *GJB2*, more than the coding portion of the *GJB6* gene itself [25].

The publication of the human genome, taken as the second landmark, was crucial for the detection of new genes associated with HL, doubling the rate of discovery from the previous period, and marking the beginning of a tendency that later improved with the combination of novel technologies, such as Next Generation Sequencing (NGS) or massive parallel sequencing, and reduced costs for NGS (Figure 1). Deafness’ high degree of related genetic heterogeneity reflects the diversity of specialized proteins required to make sense of sound and provides different points of entry into the biology of hearing. So far, genes related to HL are associated with proteins with a diverse range of functions such as adhesion, cytoskeleton, enzymes, extracellular matrix, GAP junction, ion channels, transporters, integral membrane proteins, motor, macromolecular organizers, neurosynaptic, tight junction, translation, and transcription regulators, and some with still unknown functions [26]. In addition to this etiological heterogeneity, locus heterogeneity, as in Usher syndrome [27] exemplifies the complexity of the endeavor. Several efforts to curate the HL-related gene list were made. The Hereditary Hearing Loss and Deafness Homepage [28] listed a total of 124 independent non-syndromic causative genes by August 2021 (last actualization), categorized into 77 with recessive inheritance, 51 with dominant inheritance, and 5 linked to the X chromosome (some of which can cause both recessive and dominant hearing impairment). The Clinical Genome Resource (ClinGen: https://clinicalgenome.org/, accessed on 25 January 2024) Hearing Loss Clinical Domain Working Group expert has developed a semiquantitative framework to assign clinical validity to gene-disease relationships and reported the curation of 164 HL disease gene pairs [24,25]. The GeneReviews^®^ entitled Genetic Hearing Loss Overview by Eliot Shearer et al., initially published in 1999, with a last update in September 2023, have up to date information about deafness in general and a current list of genes related to hearing loss [29]. In addition, links to the National Institute of Health (NIH), Genetic Testing Registry [30], are included for each gene. 

The genetic diagnosis of HL in the genomic age is possible, with current and emerging technologies fast tracking the diagnosis and prevention of hearing loss, for example, by rapidly identifying children susceptible to antibiotic-induced HL [31]. Molecular genetic testing is the standard of care in the evaluation of individuals with HL recommended by the American College of Medical Genetics and Genomics (ACMG) in the United States [32] and guidelines have been developed to facilitate the diagnosis. Gene panels, allowing for the simultaneous evaluation of numerous genes or genetic variants in a single assay were used. Specific panels typically confined their sequencing to a predetermined set of genes, chosen and selected by the operator, with drawbacks, such as their inability to identify variants in genes excluded from the panel, the exclusion of intronic variants, and the necessity to reanalyze negative samples as new hearing loss-related genes were discovered [33,34]. Modern genetic panels employ next-generation sequencing technologies, enabling highly efficient massively parallel sequencing, which yields significantly faster and more complete results compared to traditional methods. Confirmation tests are subsequently conducted for any identified variants using Sanger sequencing to facilitate testing in other family members. In addition, whole exome and whole genome sequencing approaches are the preferred method, when possible, since, by sequencing all genes, they have the potential to identify variants in any region [35]. 

The genetic, genomic, and clinical data accumulated is readily accessible through databases such as ClinVar and the Human Gene Mutation Database, both of which constantly curate the fast growing volume of reported genetic variants. The Deafness Variation Database is a deafness-specific open-access resource for clinical and research use that integrates all available genetic, genomic, and clinical data for research and clinical use [36]. Moreover, the Clinical Domain Working Group of the Hearing Loss Clinical Genome Resource (ClinGen) has adapted the ACMG/AMP guidelines for the classification of genetic variants within the context of HL [37]. All these databases and efforts improve the effectiveness of genetic testing, however, racial/ethnic disparities for diagnostic efficacy are well described [38].

## 3. Importance of Early Detection of Genetic Causes of Deafness 

Estimates of the global costs of hearing loss indicate that they go well beyond health and education, with exclusion from the labor force and poor quality of life being major contributors [39]. Hearing loss is on the rise worldwide. Failing to address the requirements to mitigate the number and severity of cases would not only result in increased financial burdens on healthcare systems, but also negatively impact individuals through social isolation and heightened poverty, and have broader consequences on society, leading to decreased overall productivity.

Newborn hearing screening is a widely practiced procedure worldwide, serving as a crucial tool in early identification, diagnosis, and intervention for hearing-related issues. Nevertheless, there is a subset of neonates who may pass the initial newborn hearing screening but subsequently exhibit delayed-onset and progressive hearing loss or increased vulnerability to ototoxic medications [40]. In a study performed in Beijing with parallel hearing and genetic screening performed on 180,469 newborns 25% of infants had pathogenic variants of *GJB2* or SLC26A4 and passed the routine newborn hearing screening [41]. Simultaneous screening for both hearing and genetic factors in newborns is anticipated to assume pivotal roles. This dual screening approach not only enhances the early detection and diagnosis of congenital deafness, prompting timely intervention, but also holds the potential to predict the emergence of late-onset and progressive HL. Furthermore, it aids in the identification of individuals at risk of hearing loss induced by pharmaceutical agents. While the Universal Newborn Hearing Screening (UNHS) is recognized as an immensely successful global public health initiative, it does possess certain limitations, since not all forms of hearing loss can be promptly identified through screening immediately after birth due to delayed onset. HL can have a significant impact on an individual’s ability to communicate, their overall quality of life, and their educational attainment [42], and early detection represents one of the most promising ways to reduce SNHL sequelae and to promote language and cognitive development [43]. This involves screening and timely interventions during childhood, which can also help avoid the administration of harmful medications in high-risk situations [2]. In addition, there are studies indicating that speech acquisition, auditory performance, and academic achievement are better if cochlear implants are provided before 3 years of age if needed [44,45,46,47].

Determining the genetic cause of hearing loss provides vital information, including prognosis. For example, some studies indicate that affected individuals carrying two truncating/nonsense variants in *GJB2* tend to have a more severe degree of hearing loss compared to those with two missense variants [48,49]. Additionally, patients with genetic variants in *GJB2* exhibit excellent speech perception/production skills after cochlear implantation [48]. Genetic testing can also indicate whether hearing loss is expected to be progressive or non-progressive. In addition, since ~30% of genetic HL is syndromic, genetic testing enables not only the identification of candidates for cochlear implants but also helps to pinpoint possible comorbidities early. Seeking an early diagnosis based on precise data reduces unnecessary investigations, accelerates referral for specialty care, and promotes timely treatment. Incorporating genomic sequencing improves clinical diagnosis and decreases the time to early intervention efforts and treatment [32,50].

Genetic testing also provides the opportunity of genetic counseling regarding the chance of recurrence a fundamental information for family planning. Moreover, it was reported that for patients and their families, just knowing the etiology of HL provides psychological and emotional well-being [51].

In the era of precision medicine, a model for healthcare delivery that relies heavily on data, analytics, and information, the accessibility to genetic testing is crucial [52]. Ensuring a precise and accurate molecular diagnosis is necessary to improve clinical management and for the implementation of effective surveillance and prevention initiatives. The ability to develop a more precise etiological understanding of many human conditions has sparked greater hope in improving our comprehension of the genetic causes of deafness, enhancing the prevention of this condition, and envisioning personalized therapies for patients [53,54]. To carry out efficient precision medicine, it is essential that the patient can provide their data through informed consent and that these data are accumulated in repositories so that researchers can access them and carry out an adequate analysis that allows them to identify not only the cause of that hearing loss but also the development of auditory rehabilitation [55], and eventually, the possibility of personalized treatment. Genetic testing for pediatric HL has changed the approach to clinical evaluation; however, there is still a great deal of work to be done to expand its use [56].

## 4. Individualized Prevention and Treatment for SNHL

The prevention of hearing loss is crucial at every stage of life, including prenatal, perinatal, and older age. Recent global data reports that ~34 million children have HL, and almost 60% of them result from preventable causes that can be addressed through the application of public health measures. [2]. Genetic testing and counseling are the foundation for the prevention of HL. Apart from genetic counseling for family planning, one well-known example is drug-induced ototoxicity. Aminoglycosides are broad-spectrum antibiotics that can lead to side effects such as nephrotoxicity, vestibulotoxicity, and ototoxicity [57]. One of the first discoveries in HL genetics was that aminoglycoside-induced ototoxicity clustered within families, and this trait was maternally inherited, consistent with mitochondrial inheritance. Additional studies have demonstrated that this susceptibility is caused by a variant in the 12S rRNA (RNR1) m.1555A > G [13] that induces a change in the conformation of the 12S rRNA, resulting in a structure similar to bacterial 16S rRNA, having a reported prevalence of 0.2% (approximately 1 in 500). Since aminoglycosides, in conjunction with a β-lactam, are the first-choice antibiotic for the treatment of sepsis in the neonatal period, the implementation of a rapid point-of-care test (POCT), could facilitate tailored antibiotic prescription to avoid HL [31,58].

Molecular therapies such as gene replacement, gene suppression, antisense oligonucleotides, RNA interference, and CRISPR-based gene editing [59,60,61,62], as well as precise delivery methods, techniques, and viral vectors employed for inner ear gene therapy are actively under investigation [63,64,65,66,67]. Gene therapy for HL is actively growing, and exciting new gene-therapy-based strategies to restore and prevent SNHL are arising [59] with proof-of-principle studies demonstrating the therapeutic potential of molecular agents delivered to the inner ear to treat or ameliorate different types of SNHL [68,69]. These advancements are rapidly paving the way for basic science research discoveries to transition to clinical trials, with genetic testing being foundational for the development of novel personalized therapeutic options. By late 2023, at least three separate clinical trials have been approved for DFNB9: OTOF-GT by Sensorion [70] https://www.sensorion.com/en/our-approach/restore-treat-prevent/ (accessed on 25 January 2024), AK-OTOF by Akouos, and DB-OTO by Regeneron Pharmaceuticals, Inc. (previously Decibel Therapeutics). Recently, Regeneron Pharmaceuticals, Inc. announced preliminary results showing that gene therapy improves auditory responses in a child (<2 years of age) with profound genetic HL related to variations in the otoferlin gene [71]. These are exciting new developments, both in basic science and in clinical trials with numerous challenges such as safety and longevity of the treatment, critical therapeutic time windows, and treatment efficiency 

## 5. Strengths and Barriers to Genetic Testing in HL

The strength of personalized medicine resides in a patient’s specific information regarding their condition. This aligns with the clinical duty to ensure that benefits outweigh harms for patients, with the latter being minimized as much as possible. Personalized medicine, given its greater ability to understand the unique aspects of each disease, should establish an ever-evolving feedback system as its ethical foundation [72]. Acceptance from society is essential for this kind of medicine, not just due to its accuracy and efficacy, but also because medicine is inherently a moral domain. The issue of privacy is central to precision medicine because patients must feel confident in sharing information with their healthcare providers and be guided by recommendations that often hinge on their specific genetic variations. By ensuring this, it becomes possible to align scientific and technological advancements with the concerns, principles, and values of society [73]. In genetic testing, informed consent is of paramount importance due to the profound implications of using a patient’s genetic material. As articulated in the Nuremberg Code, the voluntary consent of the human subject is essential and serves as the foundation for upholding the primacy of human dignity. Additionally, the scrutiny of private information deserves careful attention, as the analysis of specific genetic data may affect not only the patient but also their family [73]. Precision medicine must embrace patient-centeredness and engagement, digital health, data sharing, data science, genomics, and molecular technologies to be successful [52]. The limited use, thus far, of many technologies enabling early and accurate diagnoses calls for a commitment to ethical responsibility. It’s essential to uphold integrity from data collection through the implementation of personalized treatments to ensure that these ethical concerns do not become barriers hindering early diagnosis [55].

One specific hurdle for the hearing-impaired community is infrequent or ineffective interactions within the healthcare system. Difficulties in communicating through sign language, particularly with healthcare providers, can impede access to healthcare services, as well as hinder health education, outreach efforts, and disease monitoring. Personalized medicine should play a crucial role in addressing the issue of patients not having access to appropriate treatments. With the increase in treatments that are based on precision medicine, the inclusion of individuals with hearing loss holds great promise and has the potential to significantly improve their quality of life. However, given the significant challenges faced by the deaf community in clinical settings, it is essential to gain a deeper understanding of the barriers they encounter to ensure they can access the benefits of scientific knowledge [55].

Another challenge arises from potential disagreement in the understanding of deafness as a disability. While the medical perspective sees deafness as a condition requiring treatment, members of the Deaf community may view it as an aspect of their identity and a source of pride. This divergence in views can hinder the establishment of trust between healthcare professionals (clinicians or researchers) and patients, leading some to reject or doubt the benefits of personalized medicine. In addition, HL might introduce a communication barrier between healthcare professionals and patients that needs to be narrowed [53].

The early identification of many genetic diseases relies on collaboration among different disciplines and experts. This interdisciplinary approach can sometimes complicate early diagnosis, emphasizing the need to streamline processes across multiple fields to shorten the time required for detecting potential genetic diseases. To ensure personalized medicine’s positive impact and benefit for patients, it’s vital to remove barriers that might impede patient participation in this research. Understanding their concerns and promoting inclusivity while reducing health disparities are of paramount importance. Effective communication with healthcare professionals should be prioritized, and steps should be taken to narrow the divide between those who can participate in studies and those who cannot [53]. 

## 6. Current Situation in Latin America 

**Figure 2:**
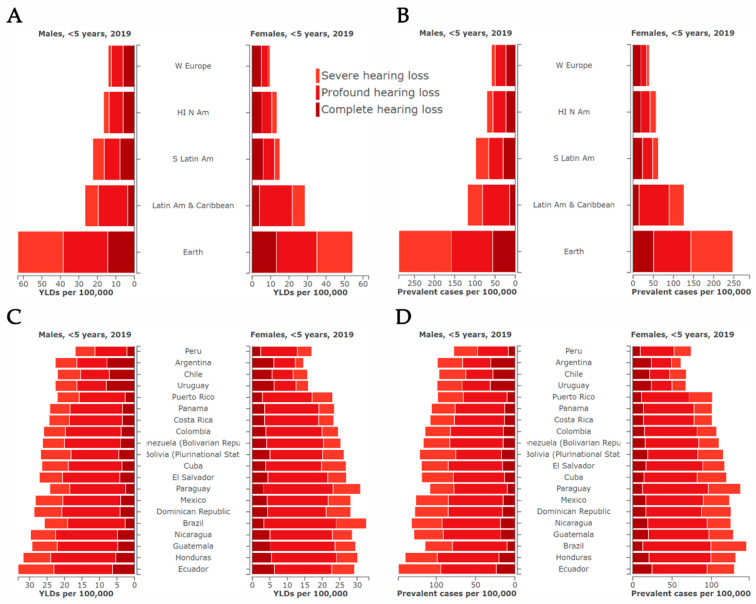
Years lived with disability (YLD) and Prevalence for early onset hearing loss. Estimates extracted from the reported data at the Global Burden of Diseases, Injuries, and Risk Factors Study (GBD) [3]. (**A**,**B**), YLD/100,000 for children <5 years of age, per region (**A**), and per country (**B**). (**C**,**D**) Prevalence/1,000,000 females or males of hearing loss in different regions (**C**) and Latin American countries. Depicted degrees of hearing loss are severe (light brown), profound (middle brown), or complete (dark brown).

Latin America is a vast region that includes Argentina, Bolivia, Brazil, Chile, Colombia, Costa Rica, Cuba, Dominican Republic, Ecuador, Guatemala, Honduras, Mexico, Nicaragua, Panama, Paraguay, Peru, Puerto Rico, El Salvador, Uruguay, and Venezuela [74]. Data regarding the frequency of hearing disorders in Latin American countries is quite limited. Estimates reported in the Global Burden of Diseases, Injuries, and Risk Factors Study (GBD) in 2019 can give an idea of YLDs and prevalence for children with severe, profound, or complete hearing loss (that at birth was attributable to congenital hearing loss). Globally, YLDs for hearing loss at <5 yo are 1.48% and at 5–14 yo 2.64%. On the bright side, Latin America, as a region, is in better shape, where for children <5 yo the YLDs is 0.8%, and for children from 5–14 yo is 1.52%, with similar prevalence and YLDs in countries throughout the region. However, in comparable high-income countries, such as Western Europe, Canada, and North America, those values are 0.63% and 0.97%, respectively (Figure 2, and Supplemental Figure S1) [3]. As these data do not discriminate between environmental and genetic causes, the higher prevalence of childhood HL is probably attributable to an increase in preventable forms of deafness, because it was reported that the relative contribution of environmental and genetic factors to the etiology of HL is highly correlated to ethnicity, the level of socio-economic development, and demographic region [3]. Although progress has been made in recent years related to prenatal and neonatal health care and immunization programs, complications within those periods and their management, are still significant causes of HL. In addition, since the use of hearing aids is calculated as an improvement in hearing level, these percentages may also reflect an earlier intervention in high-income countries [3]. There is a crucial need for extensive, well-crafted, and reliable epidemiological and prevalence research studies for Latin America, since a significant majority, around 80%, of individuals experiencing disabling hearing loss reside in countries with lower and middle incomes [2]. Encouraging and promoting these studies is essential to gain a deeper understanding of the issue and explore potential solutions. Unless action is taken, the rise of hearing impairment in the Latin American population will be exponential. WHO projections of 56 million cases by 2030 and 87 million cases by 2050 for the region raise significant concern given the barriers these countries face [75]. For example, in Argentina, based on the results of the National Study on the Profile of People with Disabilities conducted in April and May 2018 and presented by the National Institute of Statistics and Censuses (INDEC), the estimated population with hearing difficulties among children aged 6 and older is 20.8%. In this context, the distribution by severity of hearing impairment indicates that 49.0% cannot hear or use a hearing aid or cochlear implant, while 51.0% experience significant difficulty in hearing. [76,77]. In Brazil, by 2010, the incidence of hearing impairment was approximately 4 cases per 1000 births [78]. Environmental factors contribute to 80% of these cases, while genetic factors are responsible for the remaining 20% [79]. In 2022, Colombia documented 569,311 births based on data from the National Administrative Department of Statistics (DANE). Among these births, it is estimated that 2276 children (4 per 1000 births) experience permanent hearing loss [80]. In Colombia, according to information from the 2018 National Census of Population and Housing, the number of people reporting being deaf or having significant difficulty hearing was 314,320 (0.65% of the population). Among children aged 0 to 4 years, 4.8% “Cannot hear” (2.6% male and 2.2% female), and 1.4% “Can hear but with much difficulty”. The most common causes of disability for “Cannot hear” were congenital (44.3%), while 31.0% attributed it to an acquired condition. In the case of “Can hear but with much difficulty”, 12.9% were born with the condition, and 38.4% indicated that an illness caused their difficulty in hearing [81,82]. In Mexico, as of 2017, it was estimated that around 10 million people were grappling with different degrees of hearing problems. Among them, 200,000 to 400,000 individuals were experiencing complete deafness. Additionally, 2000 to 6000 children were born each year with congenital deafness. These figures highlight the significant public health issue of hearing disorders in the country during that year [83]. The World Health Organization approximates that untreated hearing loss results in a yearly worldwide expense of USD 980 billion. This encompasses expenses in the health sector (excluding the cost of hearing devices), support for education, productivity loss, and societal costs. More than half of these costs, 57%, are linked to countries with lower and middle incomes [2]. Evidence from the literature suggests that HL is not a high priority for many Latin American countries [84,85]. The UNHS is still not yet fully implemented in the region, with impairments that include limited funding, inadequate support services, shortage of qualified personnel, very long distances, and irregular distribution of both material and human resources. However, there have been improvements in the region for early detection of HL in the last few years. Argentina and Brazil have been pioneers in addressing the UNHS efforts since the 1990s. In Argentina, legislation from 2001 declares that all newborns have the right to be screened for hearing loss and to receive appropriate diagnostic evaluation and treatment. Brazil has the largest and oldest NHS programs in Latin America, with screening sites in different states. The law that determines that UNHS is mandatory for all children was approved in 2010. Colombia, which ranks among the lowest in Latin America in terms of compliance, passed the Law 1980, in 2019, granting every newborn the entitlement to undergo this crucial examination. In 2020, a global study reporting statistics for Colombia revealed that only 2.3% of newborns underwent neonatal hearing screening, clearly depicting an improvement need [86]. Some global efforts are also worth mentioning, such as the development of the hearWHO mobile and web-based app for auditory screening, intended for use across all Latin American countries, encouraging regular hearing checks, especially for those at a higher risk of hearing impairment. There’s also a professional version, hearWHOpro, tailored for health workers that enables health professionals to assess the hearing of community members, with the capacity to store extensive participant data concurrently [2] and World Hearing Day, serving as an annual global event, raising awareness about HL and advocating for ear and hearing care, with numerous Latin American countries participating [87].

Genetically speaking, the Latin American population exhibits varied genetic backgrounds, reflecting a mix of Native American, European, and African ancestry components [88]. While the ancestry of Latinos may share connections with individuals from these other regions, they represent a unique ethnic group [74], with the potential to make exciting contributions to the HL field, as adding to the array of variants within known genes, the discovery of novel inheritance mechanisms and the possibility of identifying new candidate genes. 

The genetic etiology of NSHL in Latin America was very nicely reviewed by Lezirovitz and Mingroni-Netto in 2022 [88]. Following the groundbreaking revelations about the *GJB2*/*GJB6* role, early studies on the genetics of HL in Latin America primarily concentrated on addressing the prevalence of pathogenic variants in these genes. A single chain nonsense mutation, c.35delG (NM_004004.6:c.35del; NP_003995.2: p. Gly12ValfsTer2) is the *GJB2* most frequent pathogenic variant across many different populations, with a carrier frequency as high as 1 in 31 in Caucasians from the Mediterranean [89,90,91], and accounting for up to 70% of pathologic alleles in Caucasian populations. The low frequency of c.35delG among non-European descendants, such as the Japanese or the African populations, the presence of linkage disequilibrium, and haplotype analysis suggest that it arose from a single individual 10,000 years ago, and its present distribution is the consequence of a founder event followed by migrations [91,92,93]. Approximations of the prevalence of c.35delG heterozygotes in various European nations vary between 2 to 4% within the normal hearing population. In Argentina it was found that the c.35delG mutation is the most frequent cause of NSHL, with a frequency of 1.5% in heterozygotes, a little lower than the observed among the European populations [48,94,95,96]. In Brazil, mutations in the *GJB2* gene are the leading cause of deafness in autosomal recessive inheritance, and the c.35delG mutation is the most common variant in many ethnic groups [78], where 1 in 51 Caucasians (1.9%) carries the c.35delG mutation, aligning closely with the rates observed in most European populations [97]. Studies form several regions of Brazil indicates that the c.35delG mutation is very common, with prevalence that can range from 0.97%, in neonates in the state of Sao Paulo (identified in 1 out of 103 neonates) [98], to 12.4% overall, where this mutation was identified in 23% of familial cases and 6.2% of sporadic cases [99]. Genetic studies of the *GJB2* gene from the Latin American hearing loss population confirmed the importance of *GJB2* variants as a frequent cause of non-syndromic sensorineural hearing loss, gave further support for the pathogenicity of many *GJB2* variants and contributed to the description of novel pathogenic variants [48,88,89,90,91,92,93,94,95,96,97,98,99]. Mitochondrial variants in Latin America are mostly studies of the m. 1555A > G variant [88,100,101]. Despite little information regarding the presence of other mitochondrial mutations [88], the presence of m. 1555A > G, in ~0.7% of all HL cases screened in the region makes it a good candidate for quick genetic screening to prevent drug induced ototoxicity. OTOF variants were also studied and confirmed to be an important cause of ARNSHL in the region. The studies performed in Latin America contributed to the description of novel variants, and contrary to what was observed for the GBJ2 gene, the OTOF main variant in the Spanish population, p.Gln829Ter, was not found to be very prominent [88], clearly indicating the difficulty of extrapolating variant frequencies for different populations. 

The description of genetic heterogeneity for HL within large families was previously reported in Latin America [102,103,104], as novel inheritance patterns for known genes. For example, in Brazilian families, the c.2090 T > G: (p.Leu697Trp) variant in the *MYO3A* gene was associated with AD inheritance [105,106] in contrast with the AR inheritance previously reported for this gene [28]. In addition, new candidate genes were revealed by the study of prominent Latin American families, for example, NCOA3 which was recently indicated as a candidate gene to explain autosomal dominant hearing loss in a Costa Rican family [107]. 

As mentioned above, molecular genetic testing is the standard of care in the evaluation of individuals with HL recommended by the ACMG in the United States [32], but what is the current situation in Latin America? A recent work focused on the analysis of racial and ethnic disparities in genetic testing for HL by quantifying racial/ethnic disparities. It included the analysis of 1355 populations representing 311,092 subjects from 1165 published studies. It was found that subjects of European and Asian ancestry were equivalently represented, but those of Latino American, African, and indigenous North American ancestry were significantly underrepresented. Over 96% of all subjects in the published literature were European or Asian. Specifically for Latin America, when included, most subjects derived from a small subset of countries, mainly originally from Brazil, Argentina, and Mexico [38]. The analysis of the populations included in the overall published studies over time as a function of their geographic ancestral groups showed a very reduced inclusion from Latin American populations in the analyzed period (1992–2020). When the genetic testing method for each population was observed, categorized as single-gene testing (45% of all subjects), multiple-gene testing (47%), or whole-exome sequencing (8%), the data showed that European subjects outnumbered Latin American subjects in single-gene testing reports by 18-fold, in multiple-gene testing by 250-fold, and whole-exome sequencing by 39-fold [38], depicting a huge disparity that must still be overcome. 

For a long time, most studies in the region were limited to c.35delG screening, aiming to develop affordable genetic tests with immediate relevance for diagnosis, genetic counseling, and prognosis, particularly for admixed populations with contributions of European ancestry. In most regions, even basic screenings like *GJB2*/*GJB6* are not available, and worse, numerous individuals with hearing impairment lack access to proper rehabilitation or genetic services. While Next-Generation Sequencing (NGS) is widely utilized in developed nations for the molecular diagnosis of HL, its adoption in Latin America remains far from optimal. Consequently, molecular HL diagnosis is not integrated into the public healthcare systems for most parts of the region. The genetic diversity of SNHL poses a substantial challenge for molecular diagnosis, especially in underdeveloped and genetically heterogeneous Latin American nations. In addition, the significance of each gene or genetic factor may vary significantly among diverse Latin American populations. Pinpointing the most frequently altered genes and variants in Latin America holds the potential to guide the prioritization of screenings, fast-tracking the development of more affordable and efficient screening strategies with direct applications in genetic counseling [48,89]. In Latin America the distribution and access to genomic medicine and innovations faces significant barriers beyond mere access to technology, including all previously mentioned barriers as well as the lack of trained genetic counselors. The most evident deficiency in healthcare system capacity lies in the shortage of human resources. In low-income countries for instance, nearly 78% have fewer than one ear, nose, and throat specialist per million people. Additionally, 93% lack one audiologist per million, only 17% have one or more speech therapists per million, and 50% have one or more teachers for the deaf per million. Even in countries with a relatively high number of professionals in ear and hearing care, uneven distribution and other factors can hinder accessibility. This not only presents challenges for those in need of care, but also places undue burdens on the professionals providing these services [108]. As discussed by Mitropoulos K. et al. [109], genomic medicine implementation cannot adhere to “one-size-fits-all-countries” and, as a resource-limited region, recognizing an individual country’s pressing public health priorities and disease burdens will be the way to go. Increased genetic testing, as well as increased inclusion in genetic hearing-loss studies are needed to reduce the regional disparity in diagnostic efficacy of comprehensive genetic testing. In addition, the prevalence of genetic impact and genetic factors associated with hearing impairment must be addressed to improve the unmet healthcare needs. The identification of new genetic variants associated with hearing loss in Latin America populations will pave the way for the development of effective screening tools and therapeutic strategies to either cure or slow down the progression of this condition [110,111].

## 7. Conclusions

Persistence in researching potential methods to prevent hearing loss is crucial, as it has the potential to profoundly impact a patient’s life and contribute to reducing the impact of this condition on future generations. There is no doubt that advances in personalized medicine will play a significant role in developing highly promising preventive measures, with significant progress in personalized treatment. However, it is crucial to include participants from Latin American backgrounds in genetic studies to narrow the existing gap in our understanding of the prevalence, impact, associated genetic factors, and unmet healthcare needs. 

Ensuring equitable access to the health system for hearing loss in Latin America is not an easy or short-term task. There are multiple changing variables and urgent and structural issues within the social, economic, and cultural context. Nevertheless, changes are happening worldwide in treatment, diagnosis, and communication, and Latin America should be included. To reduce the access gap, initiatives should encompass comprehensive awareness campaigns, with affordable and accessible hearing screening programs, and educational outreach to promote ear health practices. Community involvement is a crucial part of research and outreach is key. Conducting studies that include diverse patient populations representative of Latin America will contribute significantly to the understanding of regional factors influencing hearing health.

**Figure 3:**
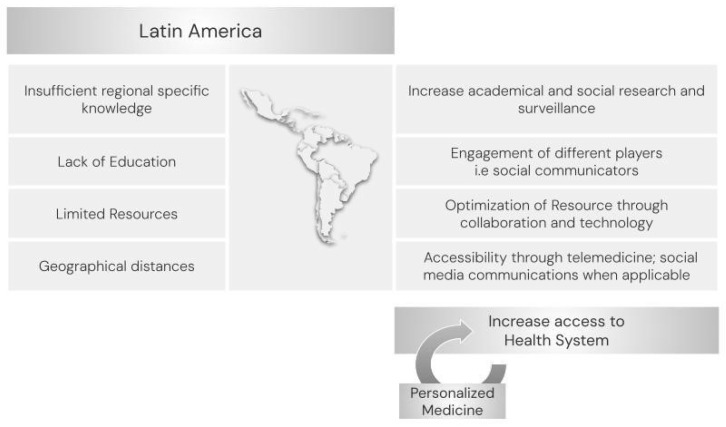
Needs and postulated alternatives for Latin America to increase access to the health system and personalized medicine.

Latin America, as a region, needs education, development, and the implementation of genomic medicine ethically and cleverly, maximizing resource usage, to reduce disparities fully and safely (Figure 3). By prioritizing inclusivity in research, we can tailor interventions to the specific needs of the population and work towards reducing the burden of hearing loss across the region. Leveraging technology, social media, and telehealth services can further enhance accessibility to hearing care, fostering a more equitable and sustainable approach to addressing this public health challenge. Involving social sciences, and communication, to provide guidance and support may ease the limitation of insufficient resources and lack of accessibility to medical health care and personalized medicine in the region.

## Data Availability

No data was used for the research described in the article.

## Supplementary Materials

The following supporting information can be downloaded at: https://www.mdpi.com/article/10.3390/genes15020178/s1, Figure S1: Years lived with disability (YLD) and Prevalence for 5–14 years of age.

## References

[1] Morton C.C., Nance W.E. (2006). Newborn hearing screening—A silent revolution. N. Engl. J. Med..

[2] Deafness and Hearing Loss|World Health Organization. https://www.who.int/health-topics/hearing-loss#.

[3] GBD 2017 Disease and Injury Incidence and Prevalence Collaborators (2018). Global, regional, and national incidence, prevalence, and years lived with disability for 354 diseases and injuries for 195 countries and territories, 1990–2017: A systematic analysis for the Global Burden of Disease Study 2017. Lancet.

[4] Report of the Informal Working Group on Prevention of Deafness and Hearing Impairment Programme Planning|World Health Organization. http://www.who.int/iris/handle/10665/58839.

[5] Zahner T. (2011). The Differential Diagnosis of Hearing Loss. Dtsch. Arztebl. Int..

[6] Marie Tharpe A.M. (2008). Unilateral and mild bilateral hearing loss in children: Past and current perspectives. Trends Amplif..

[7] Roizen N.J. (2003). Nongenetic causes of hearing loss. Ment. Retard. Dev. Disabil. Res. Rev..

[8] Van Beeck Calkoen E.A., Engel M.S.D., van de Kamp J.M., Yntema H.G., Goverts S.T., Mulder M.F., Merkus P., Hensen E.F. (2019). The etiological evaluation of sensorineural hearing loss in children. Eur. J. Pediatr..

[9] Smith R.J.H., Bale J.F., White K.R. (2005). Sensorineural hearing loss in children. Lancet.

[10] Morton N.E. (1991). Genetic epidemiology of hearing impairment. Ann. N. Y. Acad. Sci..

[11] Van Camp G., Willems P.J., Smith R.J. (1997). Nonsyndromic hearing impairment: Unparalleled heterogeneity. Am. J. Hum. Genet..

[12] Hildebrand M.S., Kahrizi K., Bromhead C.J., Shearer A.E., Webster J.A., Khodaei H., Abtahi R., Bazazzadegan N., Babanejad M., Nikzat N. (2010). Mutations in TMC1 are a common cause of DFNB7/11 hearing loss in the Iranian population. Ann. Otol. Rhinol. Laryngol..

[13] Prezant T.R., Agapian J.V., Bohlman M.C., Bu X., Öztas S., Qiu W.-Q., Arnos K.S., Cortopassi G.A., Jaber L., Rotter J.I. (1993). Mitochondrial ribosomal RNA mutation associated with both antibiotic-induced and non-syndromic deafness. Nat. Genet..

[14] Torroni A., Cruciani F., Rengo C., Sellitto D., López-Bigas N., Rabionet R., Govea N., López de Munain A., Sarduy M., Romero L. (1999). The A1555G mutation in the 12S rRNA gene of human mtDNA: Recurrent origins and founder events in families affected by sensorineural deafness. Am. J. Hum. Genet..

[15] De Kok Y.J., Van der Maarel S.M., Bitner-Glindzicz M., Huber I., Monaco A.P., Malcolm S., Pembrey M.E., Ropers H.H., Cremers F.P. (1995). Association between X-linked mixed deafness and mutations in the POU domain gene POU3F4. Science.

[16] Phippard D., Lu L., Lee D., Saunders J.C., Crenshaw E.B. (1999). Targeted mutagenesis of the POU-domain gene Brn4/Pou3f4 causes developmental defects in the inner ear. J. Neurosci..

[17] Parzefall T., Shivatzki S., Lenz D.R., Rathkolb B., Ushakov K., Karfunkel D., Shapira Y., Wolf M., Mohr M., Wolf E. (2013). Cytoplasmic mislocalization of POU3F4 due to novel mutations leads to deafness in humans and mice. Hum. Mutat..

[18] Bernardinelli E., Roesch S., Simoni E., Marino A., Rasp G., Astolfi L., Sarikas A., Dossena S. (2022). Novel POU3F4 variants identified in patients with inner ear malformations exhibit aberrant cellular distribution and lack of SLC6A20 transcriptional upregulation. Front. Mol. Neurosci..

[19] Denoyelle F., Weil D., Maw M.A., Wilcox S.A., Lench N.J., Allen-Powell D.R., Osborn A.H., Dahl H.-H.M., Middleton A., Houseman M.J. (1997). Prelingual deafness: High prevalence of a 30delG mutation in the connexin 26 gene. Hum. Mol. Genet..

[20] Zelante L., Gasparini P., Estivill X., Melchionda S., D’Agruma L., Govea N., Milá M., Monica M.D., Lutfi J., Shohat M. (1997). Connexin26 mutations associated with the most common form of non-syndromic neurosensory autosomal recessive deafness (DFNB1) in Mediterraneans. Hum. Mol. Genet..

[21] Del Castillo I., Moreno-Pelayo M.A., del Castillo F.J., Brownstein Z., Marlin S., Adina Q., Cockburn D.J., Pandya A., Siemering K.R., Chamberlin G.P. (2003). Prevalence and evolutionary origins of the del(*GJB6*-D13S1830) mutation in the DFNB1 locus in hearing-impaired subjects: A multicenter study. Am. J. Hum. Genet..

[22] Del Castillo F.J., Rodríguez-Ballesteros M., Alvarez A., Hutchin T., Leonardi E., De Oliveira C.A., Azaiez H., Brownstein Z., Avenarius M.R., Marlin S. (2005). A novel deletion involving the connexin-30 gene, del(*GJB6*-D13S1854), found in trans with mutations in the *GJB2* gene (connexin-26) in subjects with DFNB1 non-syndromic hearing impairment. J. Med. Genet..

[23] Wilch E., Zhu M., Burkhart K.B., Regier M., Elfenbein J.L., Fisher R.A., Friderici K.H. (2006). Expression of *GJB2* and *GJB6* is reduced in a novel DFNB1 allele. Am. J. Hum. Genet..

[24] Feldmann D., Le Maréchal C., Jonard L., Thierry P., Czajka C., Couderc R., Ferec C., Denoyelle F., Marlin S., Fellmann F. (2009). A new large deletion in the DFNB1 locus causes nonsyndromic hearing loss. Eur. J. Med. Genet..

[25] DiStefano M.T., Hemphill S.E., Oza A.M., Siegert R.K., Grant A.R., Hughes M.Y., Cushman B.J., Azaiez H., Booth K.T., Chapin A. (2019). ClinGen expert clinical validity curation of 164 hearing loss gene-disease pairs. Genet. Med..

[26] Dbouk H.A., Mroue R.M., El-Sabban M.E., Talhouk R.S. (2009). Connexins: A myriad of functions extending beyond assembly of gap junction channels. Cell Commun. Signal..

[27] Keats B.J.B., Savas S. (2004). Genetic heterogeneity in Usher syndrome. Am. J. Med. Genet. A.

[28] Hereditary Hearing Loss Homepage. https://hereditaryhearingloss.org/.

[29] Shearer A.E., Hildebrand M.S., Schaefer A.M., Smith R.J. Genetic Hearing Loss Overview; Updated 28 September 2023; GeneReviews®: Seattle, WA, USA. https://www.ncbi.nlm.nih.gov/books/NBK1434/.

[30] Genetic Testing Registry|National Library of Medicine. https://www.ncbi.nlm.nih.gov/gtr/.

[31] McDermott J.H., Molina-Ramírez L.P., Bruce I.A., Mahaveer A., Turner M., Miele G., Body R., Mahood R., Ulph F., MacLeod R. (2019). Diagnosing and Preventing Hearing Loss in the Genomic Age. Trends Hear..

[32] Li M.M., Tayoun A.A., DiStefano M., Pandya A., Rehm H.L., Robin N.H., Schaefer A.M., Yoshinaga-Itano C. (2022). Clinical evaluation and etiologic diagnosis of hearing loss: A clinical practice resource of the American College of Medical Genetics and Genomics (ACMG). Genet. Med..

[33] Sloan-Heggen C.M., Bierer A.O., Shearer A.E., Kolbe D.L., Nishimura C.J., Frees K.L., Ephraim S.S., Shibata S.B., Booth K.T., Campbell C.A. (2016). Comprehensive genetic testing in the clinical evaluation of 1119 patients with hearing loss. Hum. Genet..

[34] Hoefsloot L.H., Feenstra I., Kunst H.P.M., Kremer H. (2014). Genotype phenotype correlations for hearing impairment: Approaches to management. Clin. Genet..

[35] Meyts I., Bosch B., Bolze A., Boisson B., Itan Y., Belkadi A., Pedergnana V., Moens L., Picard C., Cobat A. (2016). Exome and genome sequencing for inborn errors of immunity. J. Allergy Clin. Immunol..

[36] Azaiez H., Booth K.T., Ephraim S.S., Crone B., Black-Ziegelbein E.A., Marini R.J., Shearer A.E., Sloan-Heggen C.M., Kolbe D., Casavant T. (2018). Genomic Landscape and Mutational Signatures of Deafness-Associated Genes. Am. J. Hum. Genet..

[37] Oza A.M., DiStefano M.T., Hemphill S.E., Cushman B.J., Grant A.R., Siegert R.K., Shen J., Chapin A., Boczek N.J., Schimmenti L.A. (2018). Expert specification of the ACMG/AMP variant interpretation guidelines for genetic hearing loss. Hum. Mutat..

[38] Rouse S.L., Florentine M.M., Taketa E., Chan D.K. (2022). Racial and ethnic disparities in genetic testing for hearing loss: A systematic review and synthesis. Hum. Genet..

[39] McDaid D., Park A.-L., Chadha S. (2021). Estimating the global costs of hearing loss. Int. J. Audiol..

[40] Young N.M., Reilly B.K., Burke L. (2011). Limitations of universal newborn hearing screening in early identification of pediatric cochlear implant candidates. Arch. Otolaryngol. Head Neck Surg..

[41] Dai P., Huang L.H., Wang G.J., Gao X., Qu C.Y., Chen X.W., Ma F.R., Zhang J., Xing W.L., Xi S.Y. (2019). Concurrent Hearing and Genetic Screening of 180,469 Neonates with Follow-up in Beijing, China. Am. J. Hum. Genet..

[42] Roland L., Fischer C., Tran K., Rachakonda T., Kallogjeri D., Lieu J.E.C. (2016). Quality of Life in Children with Hearing Impairment: Systematic Review and Meta-analysis. Arch. Otolaryngol. Head Neck Surg..

[43] Leroux-Roels I., Leroux-Roels G., Ofori-Anyinam O., Moris P., De Kock E., Clement F., Dubois M.-C., Koutsoukos M., Demoitié M.-A., Cohen J. (2010). Evaluation of the safety and immunogenicity of two antigen concentrations of the Mtb72F/AS02(A) candidate tuberculosis vaccine in purified protein derivative-negative adults. Clin. Vaccine Immunol..

[44] Laszig R., Aschendorff A., Beck R., Schild C., Kröger S., Wesarg T., Arndt S. (2009). Long-term functional outcomes of cochlear implants in children. HNO.

[45] Remjasz-Jurek A., Clarós P., Clarós-Pujol A., Pujol C., Clarós A. (2023). Outcomes of cochlear implantation in children with Usher syndrome: A long-term observation. Eur. Arch. Otorhinolaryngol..

[46] Lyu J., Kong Y., Xu T.-Q., Dong R.-J., Qi B.-E., Wang S., Li Y.-X., Liu H.-H., Chen X.-Q. (2019). Long-term follow-up of auditory performance and speech perception and effects of age on cochlear implantation in children with pre-lingual deafness. Chin. Med. J..

[47] Yang Y., Gao J., Du H., Geng L., Li A., Zhao N., Xu Y., Liu X., Qian X., Gao X. (2022). Influence of cochlear implants on hearing-related quality of life: Results from Chinese children with cochlear implants entering mainstream education. Int. J. Pediatr. Otorhinolaryngol..

[48] Buonfiglio P., Bruque C.D., Luce L., Giliberto F., Lotersztein V., Menazzi S., Paoli B., Elgoyhen A.B., Dalamón V. (2020). *GJB2* and *GJB6* Genetic Variant Curation in an Argentinean Non-Syndromic Hearing-Impaired Cohort. Genes.

[49] Smith R.J.H., Azaiez H., Booth K. GJB2-Related Autosomal Recessive Nonsyndromic Hearing Loss; Updated 20 July 2023; GeneReviews®: Seattle, WA, USA. https://www.ncbi.nlm.nih.gov/books/NBK1272/.

[50] Mitchell C.O., Morton C.C. (2021). Genetics of Childhood Hearing Loss. Otolaryngol. Clin. N. Am..

[51] Chari D.A., Chan D.K. (2017). Diagnosis and Treatment of Congenital Sensorineural Hearing Loss. Curr. Otorhinolaryngol. Rep..

[52] Ginsburg G.S., Phillips K.A. (2018). Precision Medicine: From Science To Value. Health Aff..

[53] Garofalo D.C., Rosenblum H.A., Zhang Y., Chen Y., Appelbaum P.S., Sabatello M. (2022). Increasing inclusivity in precision medicine research: Views of deaf and hard of hearing individuals. Genet. Med..

[54] Doo-Yi O., Byung Y.C. (2020). Genetic Information and Precision Medicine in Hearing Loss. Clin. Exp. Otorhinolaryngol..

[55] Rudman J.R., Mei C., Bressler S.E., Blanton S.H., Liu X.-Z. (2018). Precision medicine in hearing loss. J. Genet. Genom..

[56] Kenna M.A. (2022). Genetic testing for pediatric hearing loss: No time to waste. Hum. Genet..

[57] Prayle A., Smyth A.R. (2010). Aminoglycoside use in cystic fibrosis: Therapeutic strategies and toxicity. Curr. Opin. Pulm. Med..

[58] McDermott J.H., Mahood R., Stoddard D., Mahaveer A., Turner M.A., Corry R., Garlick J., Miele G., Ainsworth S., Kemp L. (2021). Pharmacogenetics to Avoid Loss of Hearing (PALOH) trial: A protocol for a prospective observational implementation trial. BMJ Open.

[59] Omichi R., Shibata S.B., Morton C.C., Smith R.J.H. (2019). Gene therapy for hearing loss. Hum. Mol. Genet..

[60] Shibata S.B., Ranum P.T., Moteki H., Pan B., Goodwin A.T., Goodman S.S., Abbas P.J., Holt J.R., Smith R.J.H. (2016). RNA Interference Prevents Autosomal-Dominant Hearing Loss. Am. J. Hum. Genet..

[61] Yoshimura H., Shibata S.B., Ranum P.T., Moteki H., Smith R.J.H. (2019). Targeted Allele Suppression Prevents Progressive Hearing Loss in the Mature Murine Model of Human TMC1 Deafness. Mol. Ther..

[62] Maeda Y., Fukushima K. (2005). In vitro and in vivo suppression of *GJB2* expression by RNA interference. Hum. Mol. Genet. Hum. Mol. Genet..

[63] Yin G., Wang X.H., Sun Y. (2023). Recent advances in CRISPR-Cas system for the treatment of genetic hearing loss. Am. J. Stem Cells.

[64] Gao X., Tao Y., Lamas V., Huang M., Yeh W.-H., Pan B., Hu Y.-J., Hu J.H., Thompson D.B., Shu Y. (2018). Treatment of autosomal dominant hearing loss by in vivo delivery of genome editing agents. Nature.

[65] Hsu P.D., Scott D.A., Weinstein J.A., Ran F.A., Konermann S., Agarwala V., Li Y., Fine E.J., Wu X., Shalem O. (2013). DNA targeting specificity of RNA-guided Cas9 nucleases. Nat. Biotechnol..

[66] Kim S., Kim D., Cho S.W., Kim J., Kim J.-S. (2014). Highly efficient RNA-guided genome editing in human cells via delivery of purified Cas9 ribonucleoproteins. Genome Res..

[67] Cradick J.T., Fine E.J., Antico C.J., Bao G. (2013). CRISPR/Cas9 systems targeting β-globin and CCR5 genes have substantial off-target activity. Nucleic Acids Res..

[68] Tao Y., Lamas V., Du W., Zhu W., Li Y., Whittaker M.N., Zuris J.A., Thompson D.B., Rameshbabu A.P., Shu Y. (2023). Treatment of monogenic and digenic dominant genetic hearing loss by CRISPR-Cas9 ribonucleoprotein delivery in vivo. Nat. Commun..

[69] Amariutei A.E., Jeng J.-Y., Safieddine S., Marcotti W. (2023). Recent advances and future challenges in gene therapy for hearing loss. R. Soc. Open Sci..

[70] Develop Innovative Therapeutic Solutions for Inner Ear Disorders|Sensorion. https://www.sensorion.com/en/our-approach/restore-treat-prevent/.

[71] Gene Therapy Improves Auditory Response for Child with Profound Genetic Hearing Loss|Contemporary Pediatrics. https://www.contemporarypediatrics.com/view/gene-therapy-improves-auditory-response-for-child-with-profound-genetic-hearing-loss.

[72] Terry Sharrer G. (2017). Personalized Medicine: Ethical Aspects. Methods Mol. Biol..

[73] Cordeiro J.V. (2014). Ethical and legal challenges of personalized medicine: Paradigmatic examples of research, prevention, diagnosis and treatment. Rev. Port. Saúde Pública.

[74] Mittal R., Patel A.P., Nguyen D., Pan D.R., Jhaveri V.M., Rudman J.R., Dharmaraja A., Yan D., Feng Y., Chapagain P. (2018). Genetic basis of hearing loss in Spanish, Hispanic and Latino populations. Gene.

[75] Hearing Loss Is on the Rise|World Health Organization. https://www.who.int/docs/default-source/documents/world-hearing-day-2018-infographic.pdf?sfvrsn=54ccef8d_12.

[76] Población con Discapacidad|Instituto Nacional de Estadística y Censos. https://www.indec.gob.ar/indec/web/Nivel4-Tema-2-21-143.

[77] Estudio Nacional Sobre el Perfil de las Personas con Discapacidad|Instituto Nacional de Estadística y Censos. https://www.indec.gob.ar/ftp/cuadros/poblacion/estudio_discapacidad_12_18.pdf.

[78] Cordeiro-SilvaI M.D.F., Barbosa A. (2010). Prevalence of 35delG/*GJB2* and del (*GJB6*-D13S1830) mutations in patients with non-syndromic deafness from a population of Espírito Santo—Brazil. Braz. J. Otorhinolaryngol..

[79] Schüffner R.d.O.A., Nascimento K.L., Dias F.A., Silva P.H.T.d., Pires W.G.B., Cipriano Junior N.M., Santos L.L. (2020). Molecular study of hearing loss in Minas Gerais, Brazil. Braz. J. Otorhinolaryngol..

[80] En Colombia, Menos del 7% de los Niños Acceden al Tamizaje Auditivo Neonatal|Sociedad Colombiana de Pediatría. https://scp.com.co/en-colombia-menos-del-7-de-los-ninos-acceden-al-tamizaje-auditivo-neonatal/.

[81] Caracterización de Ciudadanos, Usuarios y Grupos de Interés|Instituto Nacional Para Sordos. https://www.insor.gov.co/home/descargar/Caracterizacio%CC%81n-Ciudadanos-2020.pdf.

[82] CENSO Nacional de Población y Vivienda 2018|Gov.Co. https://www.dane.gov.co/index.php/estadisticas-por-tema/demografia-y-poblacion/censo-nacional-de-poblacion-y-vivenda-2018.

[83] Socorro Peña A., Contreras-Rivas A.I. (2018). Prevalencia de hipoacusia en recién nacidos sanos en un hospital de tercer nivel de atención. Detección mediante tamiz auditivo neonatal. Rev. Mex. Pediatr..

[84] Madriz J.J. (2000). Hearing Impairment in Latin America: An Inventory of Limited Options and Resources. Audiology.

[85] Gerner de Garcia B., Gaffney C., Chacon S., Gaffney M. (2011). Overview of newborn hearing screening activities in Latin America. Rev. Panam. Salud Publica.

[86] Neumann K., Euler H.A. (2020). A Survey on the Global Status of Newborn and Infant Hearing Screening. J. Early Hear. Detect. Interv..

[87] World Hearing Day|World Health Organization. https://cdn.who.int/media/docs/default-source/documents/health-topics/deafness-and-hearing-loss/world-hearing-day-2021-activity-report.pdf?sfvrsn=5710dabd_5&download=true.

[88] Lezirovitz K., Mingroni-Netto R.C. (2022). Genetic etiology of non-syndromic hearing loss in Latin America. Hum. Genet..

[89] Estivill X., Fortina P., Surrey S., Rabionet R., Melchionda S., D’Agruma L., Mansfield E., Rappaport E., Govea N., Milà M. (1998). Connexin-26 mutations in sporadic and inherited sensorineural deafness. Lancet.

[90] Gasparini P., Rabionet R., Barbujani G., Melchionda S., Petersen M., Brøndum-Nielsen K., Metspalu A., Oitmaa E., Pisano M., Fortina P. (2000). High carrier frequency of the 35delG deafness mutation in European populations. Genetic Analysis Consortium of *GJB2* 35delG. Eur. J. Hum. Genet..

[91] Tekin M., Arnos K.S., Pandya A. (2001). Advances in hereditary deafness. Lancet.

[92] Van Laer L. (2001). A common founder for the 35delG *GJB2* gene mutation in connexin 26 hearing impairment. J. Med. Genet..

[93] Gravina L.P., Foncuberta M.E., Estrada R.C., Barreiro C., Chertkoff L. (2007). Carrier frequency of the 35delG and A1555G deafness mutations in the Argentinean population. Impact on the newborn hearing screening. Int. J. Pediatr. Otorhinolaryngol..

[94] Gravina L.P., Foncuberta M.E., Prieto M.E., Garrido J., Barreiro C., Chertkoff L. (2010). Prevalence of DFNB1 mutations in Argentinean children with non-syndromic deafness. Report of a novel mutation in *GJB2*. Int. J. Pediatr. Otorhinolaryngol..

[95] Dalamón V., Béhèran A., Diamante F., Pallares N., Diamante V., Elgoyhen A.B. (2005). Prevalence of *GJB2* mutations and the del(*GJB6*-D13S1830) in Argentinean non-syndromic deaf patients. Hear. Res..

[96] Gasparini P., Gasparini P., Estivill X., Volpini V., Castellvi-Bel S., Govea N., Mila M., Della Monica M., Ventruto V., De Benedetto M. (1997). Linkage of DFNB1 to non-syndromic neurosensory autosomal-recessive deafness in Mediterranean families. Eur. J. Hum. Genet..

[97] Sartorato E.L., Gottardi E., De Oliveira C.A., Magna L.A., Annichino-Bizzacchi J.M., Seixas C.A., Maciel-Guerra A.T. (2000). Determination of the frequency of the 35delG allele in Brazilian neonates. Clin. Genet..

[98] Batissoco A.C., Abreu-Silva R.S., Braga M.C.C., Lezirovitz K., Della-Rosa V., Alfredo T., Otto P.A., Mingroni-Netto R.C. (2009). Prevalence of *GJB2* (connexin-26) and *GJB6* (connexin-30) mutations in a cohort of 300 Brazilian hearing-impaired individuals: Implications for diagnosis and genetic counseling. Ear Hear..

[99] Dalamón V., Lotersztein V., Béhèran A., Lipovsek M., Diamante F., Pallares N., Francipane L., Frechtel G., Paoli B., Mansilla E. (2010). *GJB2* and *GJB6* genes: Molecular study and identification of novel *GJB2* mutations in the hearing-impaired Argentinean population. Audiol. Neurootol..

[100] Abreu-Silva R.S., Lezirovitz K., Braga M.C.C., Spinelli M., Pirana S., Della-Rosa V.A., Otto P.A., Mingroni-Netto R.C. (2006). Prevalence of the A1555G (12S rRNA) and tRNASer(UCN) mitochondrial mutations in hearing-impaired Brazilian patients. Braz. J. Med. Biol. Res..

[101] Bezerra Salomão K., Salomão K., Ayo C. (2013). Investigation of the A1555G mutation in mitochondrial DNA (MT-RNR1) in groups of Brazilian individuals with nonsyndromic deafness and normal-hearing. Indian J. Hum. Genet..

[102] Batissoco A.C., Pedroso-Campos V., Pardono E., Sampaio-Silva J., Sonoda C.Y., Vieira-Silva G.A., da Silva de Oliveira Longati E.U., Mariano D., Hoshino A.C.H., Tsuji R.K. (2022). Molecular and genetic characterization of a large Brazilian cohort presenting hearing loss. Hum. Genet..

[103] Lezirovitz K., Pardono E., de Mello Auricchio M.T.B., de Carvalho e Silva F.L., Lopes J.J., Abreu-Silva R.S., Romanos J., Batissoco A.C., Mingroni-Netto R.C. (2008). Unexpected genetic heterogeneity in a large consanguineous Brazilian pedigree presenting deafness. Eur. J. Hum. Genet..

[104] Lezirovitz K., Nicastro F.S., Pardono E., Abreu-Silva R.S., Batissoco A.C., Neustein I., Spinelli M., Mingroni-Netto R.C. (2006). Is autosomal recessive deafness associated with oculocutaneous albinism a “coincidence syndrome”. J. Hum. Genet..

[105] Dantas V.G.L., Raval M.H., Ballesteros A., Cui R., Gunther L.K., Yamamoto G.L., Alves L.U., Bueno A.S., Lezirovitz K., Pirana S. (2018). Characterization of a novel MYO3A missense mutation associated with a dominant form of late onset hearing loss. Sci. Rep..

[106] Bueno A.S., Nunes K., Dias A.M.M., Alves L.U., Mendes B.C.A., Sampaio-Silva J., Smits J., Yntema H.G., Meyer D., Lezirovitz K. (2022). Frequency and origin of the c.2090T>G p.(Leu697Trp) MYO3A variant associated with autosomal dominant hearing loss. Eur. J. Hum. Genet..

[107] Salazar-Silva R., Dantas V.L.G., Alves L.U., Batissoco A.C., Oiticica J., Lawrence E.A., Kawafi A., Yang Y., Nicastro F.S., Novaes B.C. (2021). NCOA3 identified as a new candidate to explain autosomal dominant progressive hearing loss. Hum. Mol. Genet..

[108] World Report on Hearing|World Health Organization. https://iris.who.int/bitstream/handle/10665/339956/9789240021570-eng.pdf?sequence=.

[109] Mitropoulos K., Cooper D.N., Mitropoulou C., Agathos S., Reichardt J.K.V., Al-Maskari F., Chantratita W., Wonkam A., Dandara C., Katsila T. (2017). Genomic Medicine without Borders: Which Strategies Should Developing Countries Employ to Invest in Precision Medicine? A New “Fast-Second Winner” Strategy. OMICS A J. Integr. Biol..

[110] Peart L.S., Gonzalez J., Morel Swols D., Duman D., Saridogan T., Ramzan M., Zafeer M.F., Liu X.Z., Eshraghi A.A., Hoffer M.E. (2023). Dispersed DNA variants underlie hearing loss in South Florida’s minority population. Hum. Genom..

[111] Florentine M.M., Rouse S.L., Stephans J., Conrad D., Czechowicz J., Matthews I.R., Meyer A.K., Nadaraja G.S., Parikh R., Virbalas J. (2022). Racial and ethnic disparities in diagnostic efficacy of comprehensive genetic testing for sensorineural hearing loss. Hum. Genet..

