# Supplementary material for: Hearing Loss: Genetic Testing, Current Advances and the Situation in Latin America
Source: Genes (Basel). 2024 Jan 29;15(2):178. doi: 10.3390/genes15020178 (PMC10888486; doi:10.3390/genes15020178)
Supplement: Supplementary file 1 [file genes-15-00178-s001.zip › genes-2820841-supplementary.pdf]

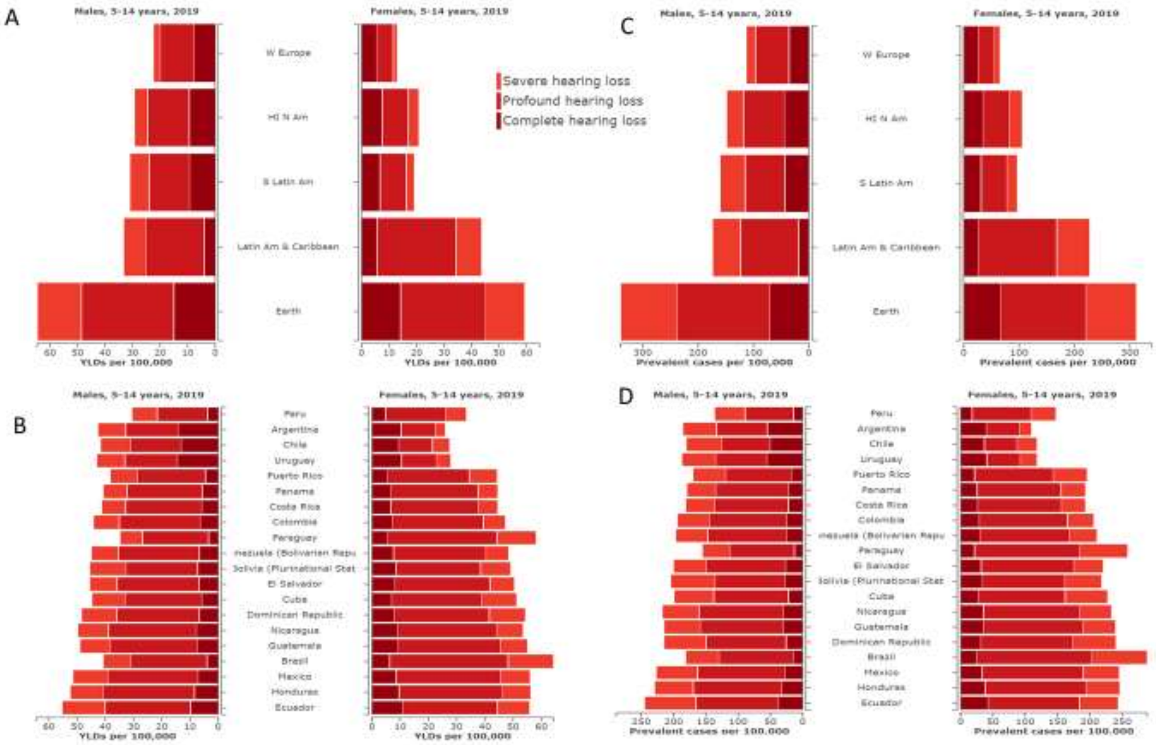

Supplemental Figure S1. *Years lived with disability (YLD) and Prevalence for 5 -14 years of age.* Estimates extracted from the reported data at the Global Burden of Diseases, Injuries, and Risk Factors Study (GBD) [3]. **A. B**, YLD/100,000 per region (A), and per country (B). **C. D**, Prevalence/1000000 females or males of Hearing loss in different regions (C) and Latin America countries. Depicted degrees of hearing loss are Severe (light brown), profound (middle brown), or complete (dark brown).
